# Supplementary material for: Variant-Specific Analysis Reveals a Novel Long-Range RNA-RNA Interaction in SARS-CoV-2 Orf1a
Source: Int J Mol Sci. 2022 Sep 21;23(19):11050. doi: 10.3390/ijms231911050 (PMC9570297; doi:10.3390/ijms231911050)
Supplement: Supplementary file 1 [file ijms-23-11050-s001.zip › ijms-1873228-supplementary.pdf]

Table S1. Number of Sequences from each Country.

Country	Number of Sequences
France	8593
Italy	3296
USA	2296
Mexico	2269
Philippines	1787
Belgium	1346
Estonia	1214
Bulgaria	1161
Czech Republic	1131
Austria	1059
India	1019
Romania	859
Spain	824
Cambodia	717
Malaysia	575
Reunion	434
Hong Kong	409
Costa Rica	365
Mayotte	354
Lithuania	307
Slovakia	258
Indonesia	244
Iran	182
Botswana	180
Japan	163
Canada	125
Vietnam	96
Switzerland	94
Bangladesh	89
Brazil	85
Bosnia and Herzegovina	77
Poland	69
South Africa	69
Zambia	61
Ghana	54
Guadeloupe	49
Nigeria	47
Iraq	45
Kenya	40
Monaco	35
Singapore	35
Russia	34
United Arab Emirates	33
French Guiana	31

Uzbekistan	31
Kosovo	30
Kuwait	30
Puerto Rico	28
Thailand	28
Maldives	27
Ukraine	27
China	25
Pakistan	24
Turkey	23
North Macedonia	22
Taiwan	17
Ireland	16
Mauritius	15
Brunei	12
Saudi Arabia	12
Togo	12
Gambia	11
Martinique	11
Montenegro	11
Germany	9
Malawi	8
Slovenia	7
Hungary	6
Nepal	6
Sri Lanka	6
Andorra	5
Cameroon	4
Chile	4
Guatemala	4
Tunisia	4
Colombia	3
Greece	3
Morocco	3
Serbia	3
South Korea	3
Ecuador	2
Saint Martin	2
Argentina	1
Belarus	1
Benin	1
Cabo Verde	1
Canary Islands	1
Egypt	1
Jordan	1
Moldova	1

Nicaragua	1
Oman	1
